# Effect of Linear Energy Transfer on Cystamine’s Radioprotective Activity: A Study Using the Fricke Dosimeter with 6–500 MeV per Nucleon Carbon Ions—Implication for Carbon Ion Hadrontherapy

**DOI:** 10.3390/molecules28248144

**Published:** 2023-12-18

**Authors:** Samafou Penabeï, Esteban Sepulveda, Abdullah Muhammad Zakaria, Jintana Meesungnoen, Jean-Paul Jay-Gerin

**Affiliations:** Département de Médecine Nucléaire et de Radiobiologie, Faculté de Médecine et des Sciences de la Santé, Université de Sherbrooke, 3001, 12ème Avenue Nord, Sherbrooke, QC J1H 5N4, Canada; samafou.penabei@usherbrooke.ca (S.P.); jesepulvedaz@gmail.com (E.S.); abdullahmuhammadzakaria@gmail.com (A.M.Z.); jintana.meesungnoen@usherbrooke.ca (J.M.)

**Keywords:** cystamine, radioprotector/antioxidant, aerated aqueous ferrous sulfate (Fricke) dosimeter, high-energy carbon ions, linear energy transfer (LET), radiolysis, chemical yields (*G* values), Monte Carlo track chemistry simulations, hadrontherapy

## Abstract

(1) Background: Radioprotective agents have garnered considerable interest due to their prospective applications in radiotherapy, public health medicine, and situations of large-scale accidental radiation exposure or impending radiological emergencies. Cystamine, an organic diamino–disulfide compound, is recognized for its radiation-protective and antioxidant properties. This study aims to utilize the aqueous ferrous sulfate (Fricke) dosimeter to measure the free-radical scavenging capabilities of cystamine during irradiation by fast carbon ions. This analysis spans an energy range from 6 to 500 MeV per nucleon, which correlates with “linear energy transfer” (LET) values ranging from approximately 248 keV/μm down to 9.3 keV/μm. (2) Methods: Monte Carlo track chemistry calculations were used to simulate the radiation-induced chemistry of aerated Fricke–cystamine solutions across a broad spectrum of cystamine concentrations, ranging from 10^−6^ to 1 M. (3) Results: In irradiated Fricke solutions containing cystamine, cystamine is observed to hinder the oxidation of Fe^2+^ ions, an effect triggered by oxidizing agents from the radiolysis of acidic water, resulting in reduced Fe^3+^ ion production. Our simulations, conducted both with and without accounting for the multiple ionization of water, confirm cystamine’s ability to capture free radicals, highlighting its strong antioxidant properties. Aligning with prior research, our simulations also indicate that the protective and antioxidant efficiency of cystamine diminishes with increasing LET of the radiation. This result can be attributed to the changes in the geometry of the track structures when transitioning from lower to higher LETs. (4) Conclusions: If we can apply these fundamental research findings to biological systems at a physiological pH, the use of cystamine alongside carbon-ion hadrontherapy could present a promising approach to further improve the therapeutic ratio in cancer treatments.

## 1. Introduction

Cancer remains a pressing global health issue, accounting for a substantial number of deaths worldwide. Yet, recent progress in its detection and treatment has resulted in improved outcomes for many patients. Of the different treatment options available, radiation therapy (RT) has proven to be a particularly effective approach for treating cancer [1]. Approximately 50% of all cancer patients undergo antitumor radiotherapy at some point during their treatment, which accounts for about 40% of curative cancer interventions [2]. The primary objective of radiation therapy is to inhibit the ability of cancer cells to multiply, leading to their eventual elimination. To optimize tumor control, it is crucial to deliver the maximum radiation dose while protecting the surrounding healthy tissue from radiation-induced damage. The use of radioprotective agents, which operate via a variety of mechanisms, has been advocated to lessen both acute and delayed radiation toxicities to normal tissues, subsequently decreasing patient morbidity and mortality [3,4,5,6,7]. Radioprotectors here aim to protect normal tissues without markedly affecting tumor cells. As such, they play a significant role in clinical radiotherapy as well as in nuclear medicine practices [8,9,10]. Radioprotective drugs may also serve to protect large populations during wide-spread radiation exposure events, including nuclear power plant accidents, nuclear weapon deployment, radiological terrorism, or astronauts on long-distance space exploration missions. Moreover, these drugs can benefit workers involved in the decontamination of fallout regions or radioactive accident sites [11,12].

In light of this, gaining a thorough understanding of the molecular mechanisms driving the actions of cytoprotective compounds is essential to more effectively control and optimize their biological impacts. This becomes particularly vital considering that transient radiation-induced free radicals are precursors of radiobiological damage in the intricate pathways that ultimately lead to cellular and tissue changes after irradiation. For instance, many potent radioprotectors effectively scavenge water-derived free radicals, thereby reducing their concentration in the medium and consequently safeguarding vital biological molecules like DNA, proteins, and membrane lipids [13,14,15].

### 1.1. Radiolysis of Water: Formation of Primary Radical and Molecular Products and Influence of the Quality of the Radiation

Aqueous systems have attracted substantial interest in radiobiology applications. Given that water is by far the most abundant component in cells and tissues (accounting for around 70–80% of their mass), the reactive species produced from its radiolysis play a major role in radiation-induced damage [16,17,18]. In the absence of oxygen, these include the hydrated electron (e^−^_aq_), the hydroxyl radical (^•^OH), the hydrogen radical (H^•^), the molecular hydrogen (H_2_), the hydrogen peroxide (H_2_O_2_), the hydronium ion (H_3_O^+^), the hydroxide ion (OH^−^), the oxygen atom O(^1^*D*) and ^•^O^•^(^3^*P*) in both its singlet ^1^*D* excited and triplet ^3^*P* ground states, etc. (e.g., see [19,20,21,22,23]). Among these, H_3_O^+^, ^•^OH, and e^−^_aq_ are produced in the highest concentrations [24]. Notably, ^•^OH is considered to be the primary species responsible for radiation damage to DNA through the indirect effect [16].

In the presence of oxygen, e^−^_aq_ and H^•^ atoms are rapidly converted to superoxide anion/hydroperoxyl (O_2_^•−^/HO_2_^•^) radicals, where O_2_^•−^ exists in a pH-dependent equilibrium with its conjugate acid (p*K*_a_ = 4.8 at 25 °C) [25]. Under normal irradiation conditions, where dose-rate effects are absent, individual radiation tracks essentially do not overlap and develop independently over time [26]. In this case, the quality of the radiation (i.e., the type and energy of the radiation used), a measure of which is given by the “linear energy transfer” (or LET, also referred to as “stopping power” by physicists and expressed in keV/μm) [27,28], is then considered the main determinant of the yields (or *G* values) of the various chemical species created and their initial, spatially nonhomogeneous geometrical distributions [29,30]. For low-LET radiation (e.g., Compton electrons produced by ^60^Co γ-rays, fast (e.g., MeV) electrons, or a few hundred MeV protons with typical LET values of ~0.3 keV/μm), the tracks are initially made up of strings of small, well-separated Magee-type “spurs” (“clusters” of reactive species, of spherical shape) [31,32] that develop in time without interference from the adjacent spurs. Under these conditions, the predominant effect of radiolysis is the generation of radicals. In the case of radiations of high LET, however, the average separation distance between neighboring spurs becomes so small that the string of spurs forms a dense, continuous, and axially homogeneous column (of cylindrical shape) of the species [29,30,33]. This allows more radicals to form in close proximity, promoting radical–radical combination or recombination reactions in the diffusing tracks. It follows that densely ionizing radiation results in the increased production of molecular products or the reformation of water, while reducing the yields of free radicals.

Depending on diffusion, the different radiolytic products may react within the spurs or tracks as they evolve over time, or they might escape and disperse into the bulk of the medium. In water at 25 °C, for low-LET radiations, the spur/track expansion is essentially complete by ~0.2 μs [34]. At this point in time, which marks the transition from nonhomogeneous track kinetics to homogeneous kinetics within the bulk solution, the radiation “track structure” no longer exists. Consequently, species that have escaped from spur/track reactions are now homogeneously distributed throughout the entire system [35,36]. The main reactive species remaining after the dissipation of spurs/tracks include e^−^_aq_, H^•^, and ^•^OH (the “radical” products), along with H_2_ and H_2_O_2_ (the “molecular” products). While commonly referred to as “primary” species, this designation is not entirely accurate [26]. Nevertheless, the yields of these species are frequently termed “primary” or “escape” yields, symbolized by *g*(e^−^_aq_), *g*(H^•^), *g*(^•^OH), *g*(H_2_), and *g*(H_2_O_2_). (It is worth noting that the lower-case ‘*g*’ denotes these primary yields; experimentally measured or final yields are always represented as *G*(X) [20]). Once homogeneity is reached, these species become available to react with the dissolved solutes that were present in either low or moderate concentrations during the irradiation process.

Throughout this article, radiation chemical yields are expressed in units of molecules formed (or consumed) per 100 eV of absorbed energy. For conversion into SI units (mol/J): 1 molecule/100 eV ≈ 0.10364 μmol/J [20,21,22,23].

### 1.2. Employing the Aqueous Ferrous Sulfate (Fricke) Dosimeter as an Indicator of Cystamine’s Radioprotective and Antioxidant Properties in the Context of Irradiations by Fast Carbon Ions in the Energy Range of 6–500 MeV per Nucleon

At the molecular level, chemical (i.e., nonbiological) radioprotectors for low- and high-LET ionizing radiation exert their protective effects in cellular systems through diverse mechanisms. Of particular significance are the suppression of indirect radiation damage through water-derived free-radical scavenging and the repair of direct or indirect damage through H^•^ atom transfer or donation (e.g., see [4,7,10,16]). In the first mechanism, radioprotector compounds remove or “scavenge” the highly reactive intermediates produced by water radiolysis before they can interact with and damage target biomolecules, especially DNA, thereby mitigating the harmful effects of radiation. In the second mechanism, radioprotectors with sulfhydryl (–SH) groups, known for their labile hydrogen atoms, can also provide cytoprotective action. They do so by donating H^•^ atoms, chemically repairing both direct and indirect molecular lesions in target macromolecules. This repair occurs after lesion formation but before the damage becomes irreversible due to the addition of O_2_ and the subsequent formation of peroxyl radicals, which prevent the regeneration of the original compound. In this latter scenario, sulfhydryl molecules effectively compete with oxygen for interactions with DNA free radicals, thereby minimizing DNA damage and enhancing cell viability [37].

The majority of chemical radioprotective agents that have been developed and tested are aminothiols. Cystamine (RSSR, where R = NH_2_–CH_2_–CH_2_) is the disulfide form of cysteamine (also known as 2-mercaptoethylamine or 2-aminoethanethiol, RSH), a member of the same aminothiol family, renowned for its radioprotective properties [38,39]. Depending on the local redox environment within cells, this disulfide undergoes in vivo reduction, resulting in the formation of two cysteamine molecules following the cleavage of its highly unstable disulfide bond [40,41]. The current understanding of the mechanisms by which cystamine exerts its action in vivo [42,43] suggests that cysteamine is the key intermediate involved in the radiation-protective properties of this compound.

Below pH 8, cystamine is predominantly in the form of a doubly protonated molecule, represented by the symmetric formula NH_3_^+^–CH_2_–CH_2_–S–S–CH_2_–CH_2_–NH_3_^+^ (with p*K*_a_ values of 8.7–9 for both of the –NH_3_^+^ groups) [44,45]. The mutual Coulomb repulsion between the two positively charged groups at opposite ends of the molecule promotes an open conformation with a high accessibility of the –S–S– center to approaching radicals [44]. This conformational feature is a significant determinant of this compound’s ability as a water-based free-radical scavenger, which explains its strong antioxidant profile. In addition to its role as a radical scavenger in protecting against cellular oxidative stress, cystamine also exhibits antiapoptotic properties. These properties have the potential to delay, halt, or even reverse the progression of neuronal degeneration seen in central nervous system disorders, such as Huntington’s disease, Alzheimer’s disease, and Parkinson’s disease, as demonstrated in animal models [40,41,42,43,46,47]. Cystamine has also demonstrated the ability to reduce brain swelling, cell death, and neurological deficits in rats following intracerebral hemorrhages [48]. Moreover, it has been shown to significantly suppress HIV replication in cultured lymphocytes and macrophages [49]. Nevertheless, the exact molecular mechanisms through which it operates remain unclear.

Several prior studies [50,51], our own included [13], have employed the well-known radiolytic oxidation of ferrous (Fe^2+^) to ferric (Fe^3+^) ions in the aqueous ferrous sulfate, or Fricke, chemical dosimeter [20,52,53] to evaluate the radical scavenging abilities of cystamine and, consequently, its potential as a radioprotective/antioxidant. Although the Fricke dosimeter was initially designed as a dose-measuring device, it also serves as a valuable tool at the molecular level for investigating the impact of adding any scavenger of the primary chemical species of water radiolysis on the radiolytic ferric ion, or Fricke, yield *G*(Fe^3+^) (e.g., see [54,55,56,57]). By inference, if a scavenger molecule, such as cystamine, is present in the Fricke solution during irradiation, it will competitively react with the products of the radiolysis of water before they can react with Fe^2+^, leading to a reduced yield of Fe^3+^ (i.e., there will be protection of Fe^2+^). The observed reduction in *G*(Fe^3+^) with cystamine present was further corroborated by Monte Carlo simulations of the radiolysis of Fricke–cystamine solutions, both with and without oxygen [13,14,15].

In our previous research, we initially utilized our IONLYS-IRT Monte Carlo track chemistry computer code [58,59,60] to simulate the radiolysis of Fricke–cystamine solutions by using 300 MeV incident protons, mimicking the low LET of cobalt-60 γ-rays or fast electrons [13]. These simulations covered a wide range of cystamine concentrations (10^−6^–1 M). Following that, we investigated the influence of radiation’s LET on cystamine’s radioprotective ability by adjusting the energy of the irradiating protons from 150 keV to 500 MeV. This adjustment corresponds to LET values that range from approximately 72.3 keV/μm down to 0.23 keV/μm [14]. Lastly, we investigated the influence of dose rate on *G*(Fe^3+^) by employing a multitrack irradiation model along with an extended version of our IONLYS-RT code [61]. This enabled us to simulate the radiolysis of Fricke–cystamine solutions with single and instantaneous (Dirac) pulses of 300 MeV incident protons [15]. This scenario is pertinent to the “FLASH effect” in radiobiology [62,63,64] or, for example, a nuclear power plant accident [65].

In this study, our goal is to build on our previous research to further elucidate the mechanisms that underlie the protective and antioxidant efficacy of cystamine against exposure to irradiation by fast carbon ions (^12^C^6+^ nuclei, i.e., carbon atoms stripped from all their electrons). We aim to investigate a wide energy spectrum, specifically from 6 to 500 MeV per nucleon, aligning with LET values from ~248 keV/μm to 9.3 keV/μm. Through this, we aspire to unravel the complex effectiveness of cystamine in counteracting the detrimental effects of high-energy heavy charged particles. Notably, this range encompasses the energies commonly utilized in clinical carbon ion hadrontherapy, typically around 400 MeV per nucleon. The LET is generally under 10 keV/μm at the “entrance channel” but can ascend to above 80 keV/μm within the tumor core [66], offering a significant overlap with our investigative range.

In the field of cancer treatment, especially for deep-seated and traditionally radioresistant local tumors, carbon ion hadrontherapy (currently, there are 10 centers actively treating patients with carbon ions and more under development worldwide) is recognized for its superior tumor control capabilities compared to conventional photon radiotherapy or even proton therapy [67,68,69,70,71]. This superiority is due to its enhanced sparing effects on healthy tissues and greater biological effectiveness, meaning that at a given dose of radiation, it kills tumor cells more efficiently than conventional radiation modalities [4]. These advantageous properties are attributed to the densely ionizing nature of carbon ions in the so-called “Bragg peak” region (e.g., see [72,73,74]), where they deposit most of the therapeutic dose at a specific depth within the tumor volume. Such a characteristic depth–dose distribution of ions makes carbon ion therapy highly effective in treating hypoxic (i.e., radioresistant) tumors [66].

Throughout this study, we assumed that the dose rates are low enough to avoid overlap between the tracks of different incident carbon ions. Under these conditions of a lack of dose-rate effects, the space–time history of just a single track needs to be considered. The effects of high dose rates will be examined in a later work.

## 2. Results and Discussion

### 2.1. Kinetics of Fe^3+^ Formation in Fricke–Cystamine Solutions Subjected to 6–500 MeV per Nucleon Carbon Ion Irradiation

**Figure 1:**
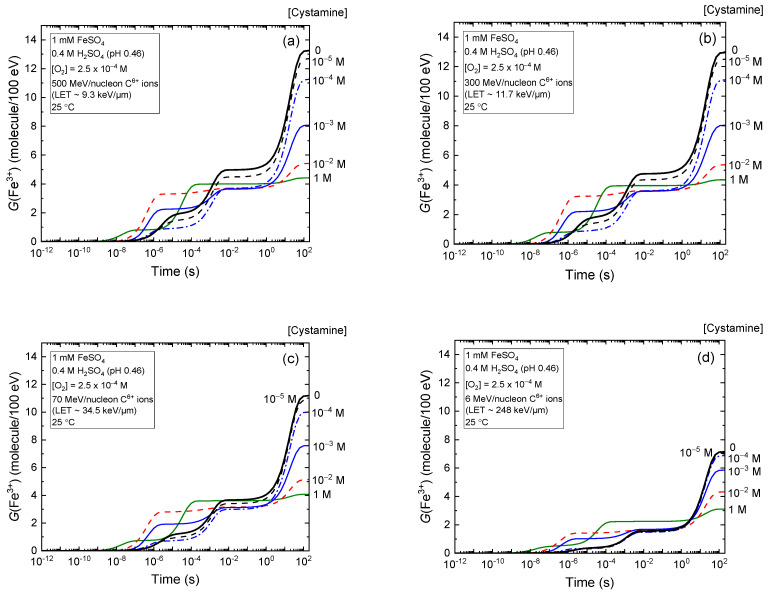
Time evolution of *G*(Fe^3+^) (expressed in molecules per 100 eV) from our Monte Carlo simulations of the radiolysis of Fricke–cystamine solutions (containing 1 mM of FeSO_4_ in aerated 0.4 M H_2_SO_4_) with varying concentrations of cystamine. We employed ^12^C^6+^ ions at incident energies of 500 MeV per nucleon (panel (**a**)), 300 MeV per nucleon (panel (**b**)), 70 MeV per nucleon (panel (**c**)), and 6 MeV per nucleon (panel (**d**)), corresponding to LET values of approximately 9.3, 11.7, 34.5, and 248 keV/μm, respectively. All calculations were conducted at a constant temperature of 25 °C. The various lines in the figures represent five different cystamine concentrations, as follows: 10^−5^ M (dashed black line), 10^−4^ M (dash-dot blue line), 10^−3^ M (solid blue line), 10^−2^ M (dashed red line), and 1 M (solid olive line). These concentrations are indicated to the right of the figures. For reference, the solid black lines show the simulated kinetics of Fe^3+^ ion formation for the Fricke dosimeter without any added cystamine, under identical irradiation conditions.

In Figure 1a–d, we examine the effect of LET on the formation kinetics of Fe^3+^ ions, as derived from our simulations of the radiolysis of aerated Fricke–cystamine solutions subjected to 6–500 MeV carbon ion irradiation, with varying concentrations of cystamine (see Section 3, “Materials and Methods”).

As can be seen, *G*(Fe^3+^) drops at ~200 s as the cystamine concentration increases, regardless of whether the source of irradiation is low-LET 500 MeV per nucleon (~9.3 keV/μm) or high-LET 6 MeV per nucleon (~248 keV/μm) carbon ions. However, this decrease is notably more significant during low-LET irradiations. Specifically, under 500 MeV per nucleon carbon ion irradiation, *G*(Fe^3+^) falls from roughly 13.3 to about 4.4 molecules per 100 eV–a 8.9 *G*-unit reduction–when comparing a Fricke solution without cystamine against one containing 1 M of the disulfide (Figure 1a). Conversely, for 6 MeV per nucleon carbon ion irradiation, *G*(Fe^3+^) decreases from about 7.2 to 3.1 molecules per 100 eV, equating to a smaller ~4.1 *G*-unit reduction (Figure 1d). These diminished Fe^3+^ ion yields in the presence of cystamine during irradiation clearly suggest that this molecule can neutralize the primary products of acid water radiolysis, mainly H^•^ atoms and ^•^OH radicals (see Section 3.2), which are responsible for the radiolytic oxidation of ferrous ions in the absence of cystamine. From a mechanistic perspective, the ability of cystamine to capture radicals underlines its potent “antioxidant profile”. This allows the compound to compete with Fe^2+^ ions for the free radicals produced during the irradiation of the surrounding water.

**Figure 2:**
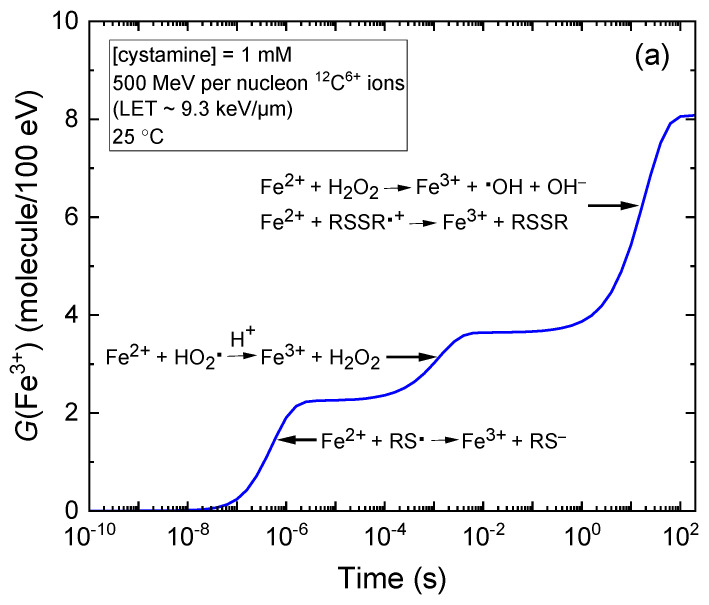

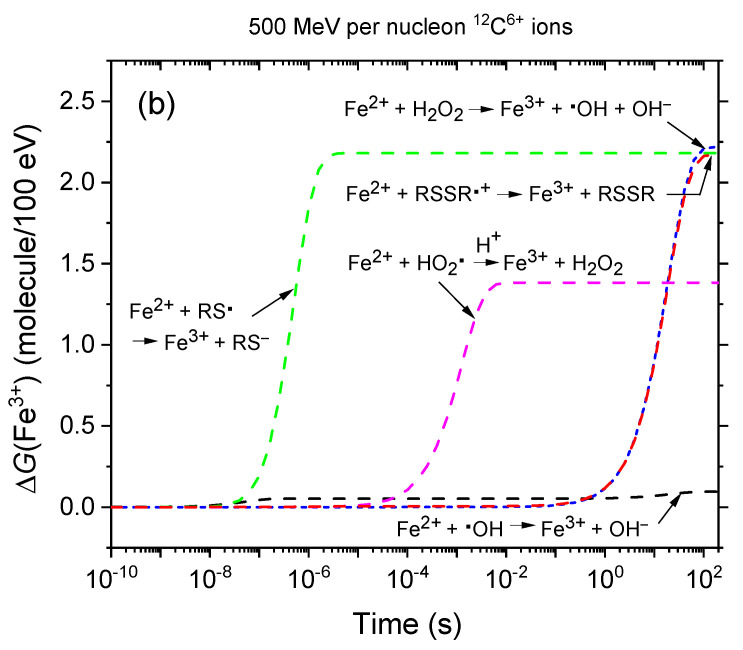
Panel (**a**) depicts the temporal evolution of *G*(Fe^3+^) expressed in molecules per 100 eV, for 500 MeV per nucleon incident carbon ions with an LET of ~9.3 keV/μm in the radiolysis of aerated Fricke dosimeter solutions. These solutions consist of 1 mM of FeSO_4_ and 1 mM of cystamine in aqueous 0.4 M H_2_SO_4_ at 25 °C. The concentration of dissolved O_2_ assumed in the simulations is 0.25 mM. The solid blue curve illustrates our simulated kinetics for the formation of Fe^3+^ ions. Panel (**b**) shows the time-dependent extents Δ*G*(Fe^3+^), expressed in molecules per 100 eV, of the various reactions contributing to the generation of Fe^3+^ ions. These outcomes, calculated via our Monte Carlo simulations over the interval from 10^−10^ to 200 s, indicate that the oxidation of Fe^2+^ to Fe^3+^ predominantly occurs through reactions with HO_2_^•^ (dashed magenta line), H_2_O_2_ (short-dashed blue line), and the radical species derived from cystamine RS^•^ and RSSR^•+^ (depicted by dashed green and red lines, respectively). Detailed mechanisms of these reactions are discussed in Section 3.

**Figure 3:**
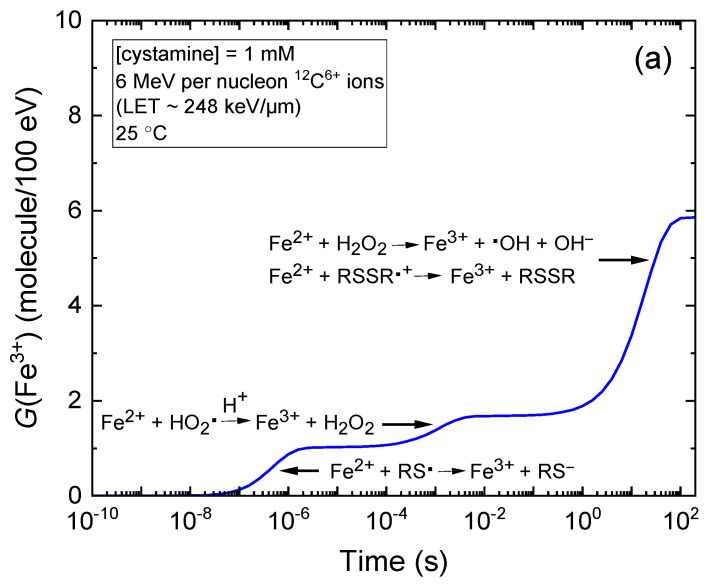

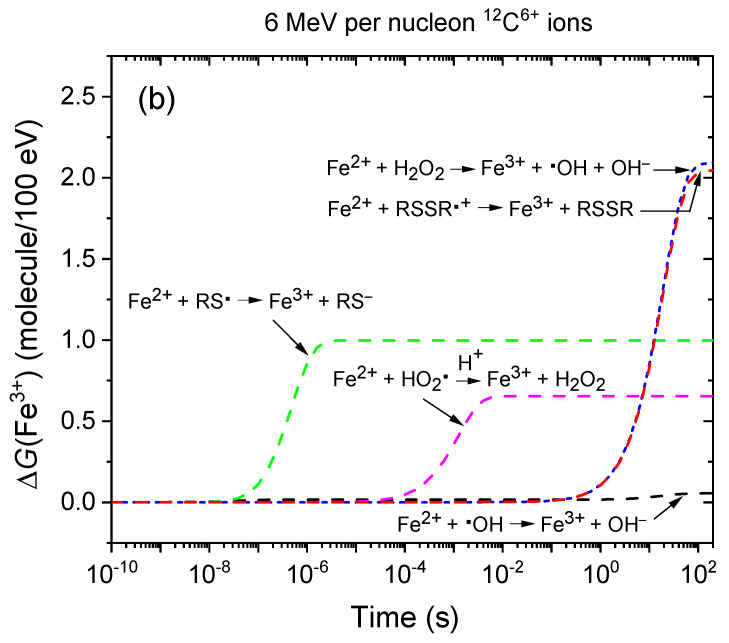
Panel (**a**) illustrates the time-dependent evolution of *G*(Fe^3+^) (in molecules per 100 eV) for 6 MeV per nucleon incident carbon ions with an LET of ~248 keV/μm in the radiolysis of aerated Fricke dosimeter solutions. These solutions are composed of 1 mM of FeSO_4_ and 1 mM of cystamine dissolved in aqueous 0.4 M sulfuric acid at 25 °C. The concentration of dissolved O_2_ used in the simulations is 0.25 mM. Similar to what is depicted in Figure 2, the solid blue curve represents our simulated kinetics of Fe^3+^ ion formation. Panel (**b**) shows the time-dependent extents Δ*G*(Fe^3+^), expressed in molecules per 100 eV, of the various reactions contributing to the generation of Fe^3+^ ions. The results, obtained from our Monte Carlo simulations covering the interval from 10^−10^ to 200 s, demonstrate that the oxidation of Fe^2+^ to Fe^3+^ mainly occurs via reactions with HO_2_^•^, H_2_O_2_, and the cystamine-derived radicals RS^•^ and RSSR^•+^ (see the caption of Figure 2 for details).

Figure 1a–d also show that *G*(Fe^3+^) is time-dependent due to the varied reaction time scales of Fe^2+^ oxidation reactions, which contribute to the formation of Fe^3+^ in the radiolysis of the Fricke–cystamine system under aerated conditions (see Section 3.2). For illustrative purposes, we selected the kinetics of Fe^3+^ formation in 1 mM of cystamine solutions exposed to incident carbon ions with energies of 500 MeV per nucleon and 6 MeV per nucleon, as depicted in Figure 2 and Figure 3, respectively. In both specified incident carbon ion energy scenarios, Figure 2a and Figure 3a show that the oxidation process of Fe^2+^ ions to Fe^3+^ predominantly occurs through reactions with HO_2_^•^, H_2_O_2_, and the cystamine-derived radical species RS^•^ and RSSR^•+^.

Notably, the most rapid reaction occurs between Fe^2+^ ions and thiyl radicals, RS^•^, which proceeds in just a few microseconds. This is clearly demonstrated in Figure 2b and Figure 3b, where we show the temporal profiles of the extents Δ*G*(Fe^3+^) pertaining to each reaction that leads to the formation of Fe^3+^, as derived from our simulations for irradiating carbon ions at energies of 500 MeV per nucleon and 6 MeV per nucleon, respectively (see Section 3). These thiyl radicals primarily originate from the (RSSR + H^•^) Reaction (11). The H^•^ atoms that remain unreacted with cystamine subsequently react with oxygen on the microsecond scale to yield HO_2_^•^. These hydroperoxyl radicals are responsible for the subsequent oxidation of Fe^2+^ ions to Fe^3+^, a process that is comparatively slower, requiring ~10 ms for completion, as illustrated in Figure 2b and Figure 3b. Ultimately, beyond ~1 s, two further reactions predominantly account for the formation of Fe^3+^. These reactions are nearly superimposed: Reaction (8), which involves H_2_O_2_ and Fe^2+^ and Reaction (20), where RSSR^•+^ reacts with Fe^2+^. Both reactions reach completion by about 200 s. Notably, these two reactions are interlinked; the H_2_O_2_ produced contributes to the yield of Fe^3+^ in Reaction (8) and also to the formation of an equivalent yield of ^•^OH radicals. In this specific scenario, with solutions containing 1 mM of Fe^2+^ ions and 1 mM of cystamine, virtually all the ^•^OH radicals so generated are scavenged by cystamine before they can react with Fe^2+^. Indeed, at the same concentration, cystamine outcompetes Fe^2+^ for ^•^OH due to the significantly higher rate constant of Reaction (12) (1.7 × 10^10^ M^−1^ s^−1^), which is 50 times larger than that of Reaction (6) (3.4 × 10^8^ M^−1^ s^−1^).

### 2.2. Effect of Cystamine Concentration on the Fricke Yield

**Figure 4:**
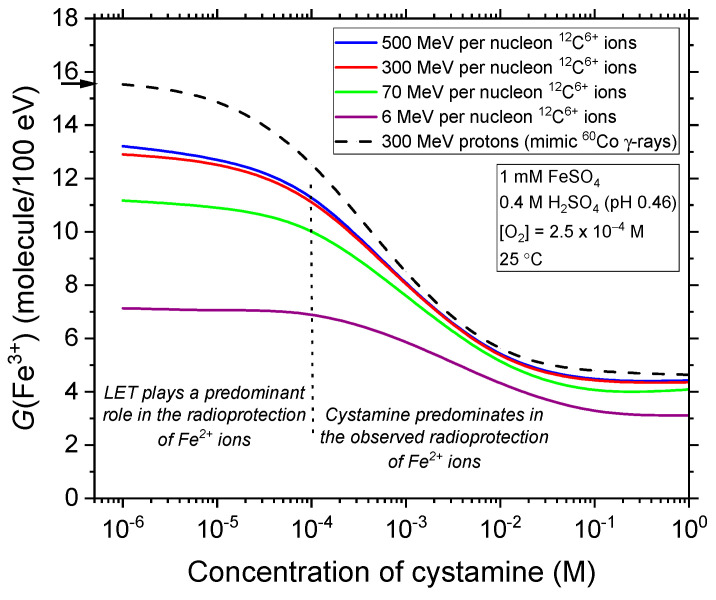
Dependence of the Fe^3+^ ion yield (in molecules per 100 eV), obtained from our Monte Carlo simulations of the carbon ion radiolysis of Fricke–cystamine solutions at ~200 s after ionization, upon the concentration of added cystamine in the range 10^−6^–1 M. These results were obtained using varying carbon ion energies, namely: 500 MeV per nucleon (LET~9.3 keV/μm), 300 MeV per nucleon (LET~11.7 keV/μm), 70 MeV per nucleon (LET~34.5 keV/μm), and 6 MeV per nucleon (LET~248 keV/μm), without considering dose-rate effects and under aerated conditions at 25 °C. The vertical dotted line at ~10^−4^ M cystamine concentration marks the shift between the two distinct radioprotective effects: the first primarily influenced by the LET itself (at concentrations < 10^−4^ M) and the second chiefly driven by the presence of cystamine (at concentrations > 10^−4^ M) (see text). For reference, the dashed black curve shows the *G*(Fe^3+^) values we calculated for 300 MeV irradiating protons, simulating the effects of ^60^Co γ-radiolysis [13,14,15]. The arrow to the left of the figure indicates the accepted value (15.5 ± 0.2 molecules per 100 eV) of the yield of the aerated Fricke dosimeter when exposed to ^60^Co γ-rays or fast electrons, without the addition of cystamine.

The effect of cystamine concentration on the Fricke yield is further illustrated in Figure 4. Here, our calculated *G*(Fe^3+^) values at ~200 s are shown for different energies (or LETs) of the irradiating carbon ions, with cystamine concentrations ranging from 10^−6^ to 1 M. From the data presented, several observations can be made.

Firstly, in the absence of cystamine, *G*(Fe^3+^) (expressed in molecules per 100 eV) decreases from 13.3 at 9.3 keV/μm to 13.0 at 11.7 keV/μm, further reducing to 11.2 at 34.5 keV/μm, and lastly, dropping to 7.2 at 248 keV/μm (see Figure 1). Remarkably, these values are in close agreement with the experimentally reported yields of Fe^3+^ ions for the Fricke dosimeter under high-energy carbon ion irradiations, as documented in studies by Christman et al. [75] and LaVerne and Schuler [76]. The marked reduction in *G*(Fe^3+^) with higher LET primarily stems from changes in the spatial distribution of radiation-induced radicals (i.e., in the “track structure”) when moving from low- to high-LET ionizing radiations [14,20,28,29,35,77,78]. In fact, this behavior can theoretically be attributed to the increased importance of intratrack processes when dealing with more densely ionizing radiations at high LET. These processes amplify radical–radical combination reactions, resulting in the formation of molecular products. Then, when a scavenger, such as Fe^2+^ ions, is present, this increased molecular product yield reduces the occurrences of radical scavenger reactions at long times. In other words, the lower the number of free radicals that manage to escape from the carbon ion tracks, the lesser the oxidation of ferrous ions. According to Equation (5), this, in turn, leads to reduced Fricke *G* values. Our results are consistent with the many experimental data found in the literature, which discuss the influence of LET on the chemistry and yields of the Fricke dosimeter (e.g., see [20,53,77,78,79]).

Secondly, Figure 4 indicates that the decrease in *G*(Fe^3+^) can be attributed to two (additive) radioprotective effects: one stemming from the effect of LET itself (as described above) and the other due to the presence of cystamine. At lower cystamine concentrations (below, say, ~10^−4^ M), cystamine exhibits limited efficacy in reducing the Fricke yield. As the LET increases, its efficiency declines gradually. Notably, *G*(Fe^3+^) becomes more or less independent of the cystamine concentration at the lowest carbon ion energy (6 MeV per nucleon, corresponding to ~248 keV/μm, our highest LET) considered. This can be easily understood by the fact that higher LET values lead to the generation of fewer radicals (the ones with which cystamine reacts) and an increased production of molecular products like H_2_O_2_, H_2_, and reconstituted water. These molecular products are largely nonreactive with cystamine. Within this concentration range of cystamine, it can thus be inferred that the significant reduction in *G*(Fe^3+^) seen in Figure 4 is primarily due to the effect of LET, rather than the presence of cystamine. In essence, LET itself acts as a radioprotector for the Fricke dosimeter solution. Conversely, at these lower cystamine concentrations, cystamine offers less protection at high LET, a conclusion previously noted in the literature (see, e.g., [4,11,80]).

Thirdly, when cystamine concentrations exceed ~10^−4^ M, there is a pronounced decrease in *G*(Fe^3+^), regardless of the LET value. This significant drop in *G*(Fe^3+^) suggests that at these high concentrations, it is the presence of cystamine, not the LET, that primarily contributes to the observed radioprotection of Fe^2+^ ions during the radiolysis of Fricke–cystamine solutions. This is because, under these conditions, cystamine progressively intervenes in the carbon ion tracks before they fully expand. Indeed, for 10^−4^ M cystamine, we can estimate the time scales during which the scavenging of e^−^_aq_, H^•^, and ^•^OH by RSSR is occurring (Reactions (10)–(12)). By using the inverse of the “scavenging power”, defined as the product *k*[RSSR] (with units of s^−1^) [20], we determined that these reactions with cystamine take place at ~0.25, 1.25, and 0.6 μs, respectively. Each of these times falls within the microsecond range, which precisely aligns with the moment when the tracks dissipate, allowing the remaining radicals to react with Fe^2+^ ions in the main body of the solution. As a result, cystamine will be able to effectively scavenge the early formed free radicals in the columnar regions of the carbon ion tracks, regions with very high radical concentrations. This activity, in competition with the inter-radical combination reactions in the radiolysis track stage, further diminishes the radicals available to oxidize Fe^2+^ ions. As the concentration of cystamine increases further, this competition between intratrack radical combination and capture by the cystamine is increasingly in favor of the cystamine–radical reactions. Consequently, at moderate to high concentrations of cystamine (above about 10^−3^ M), the production of Fe^3+^ is less and less influenced by changes in the track structure’s geometry. This, in turn, leads to a reduced LET effect at these concentrations, as shown in Figure 4.

Let us highlight two final observations. First, as can be seen in Figure 4, *G*(Fe^3+^) remains largely unchanged when the cystamine concentration exceeds ~0.1 M, regardless of the LET value. Building on previous studies related to proton irradiation under high-LET [14] and high dose-rate [15] conditions, the data suggest that even at the highest cystamine concentrations considered in this study, the compound cannot neutralize all initially-formed radicals in the high-LET carbon ion tracks quickly enough (within picoseconds or faster), thus impeding its capability to counteract the radical–radical combination reactions. Second, and of significant note, it has been reported that at high concentrations (>1 M), cystamine acts as a “dry” (nonhydrated) electron scavenger during the subpicosecond physicochemical stage of radiolysis [81]. It can capture low-energy electrons, specifically those with energies below the lowest electronic excited states of water, including subexcitation, quasi-free epithermal, and thermal electrons [82,83]. This occurs before these electrons, which, from a quantum-mechanical perspective, lie near the bottom of the lowest conduction band of water, become trapped and stabilized within their “hydrated” potential energy wells, often termed as “cages” or “cavities”, and represented as e^−^_aq_ [28]. At a concentration of 1 M, the ability of cystamine to scavenge dry electrons could significantly influence its radioprotection properties. This can readily be seen from Equations (10)–(12): the reaction of cystamine with dry electrons can decrease the number of e^−^_aq_ and, consequently, the quantity of H^•^ atoms in the irradiated Fricke solution, potentially reducing *G*(Fe^3+^) (see Equation (5)). In our current simulation model, we did not account for this dry electron scavenging property of cystamine. Yet, based on a procedure we previously developed [84], there is ongoing research aiming to evaluate how incorporating the ultrafast capture of e^−^_aq_ precursors by cystamine affects the formation of Fe^3+^ ions when the concentration of cystamine exceeds 1 M.

### 2.3. Monte Carlo Track Chemistry Simulations of the Radiolysis of Fricke–Cystamine Solutions: Effect of Multiple Ionization under 6 MeV per Nucleon (~248 keV/μm) Carbon Ion Irradiation

We now focus on understanding the impact of very high LETs on cystamine’s radioprotective and antioxidant properties, considering the multiple (primarily double) ionization (MI) of water in our study. This scenario is especially relevant in the context of 6 MeV per nucleon carbon ion irradiation, where the LET reaches ~248 keV/micron. It is important to remember that the MI mechanism is suggested to play a key role in the formation of superoxide anion/hydroperoxyl.

(O_2_^•−^/HO_2_^•^; p*K*_a_ = 4.8) radicals, as well as molecular oxygen, during the heavy ion radiolysis of liquid water at high LET [30,77,85,86,87] (for a review, see [60]). In short, our model accounts for the direct MI effects on the outer (loosely bound) electron shells of the target by incorporating double ionization processes in single-ion-water collisions. Ionizations of higher multiplicity are not included because they are much less probable at the specific LET of interest. The double ionization cross-section values for carbon ions employed in our track structure simulation modeling have previously been described in detail [30,60,88] and are therefore not discussed further here. While infrequent compared to single ionizations, these doubly ionized water molecules are “extremely effective chemically” due to their pronounced instability in solution [89]. Here, the rearrangement of these thermodynamically unstable water cations was treated according to the general mechanism suggested by Ferradini and Jay-Gerin [86], which asserts that, in liquid water, H_2_O^2+^ undergoes dissociation at very short times, driven by the overall acid–base re-equilibration reaction (see Table 14.3 in Meesungnoen and Jay-Gerin [60]):

H_2_O^2+^ + 2H_2_O → 2H_3_O^+^ + ^•^O^•^(^3^*P*), (1)

followed by

^•^OH + ^•^O^•^(^3^*P*) → HO_2_^•^, (2)

in the heavy ion track due to the very high local concentration of radicals.

**Figure 5:**
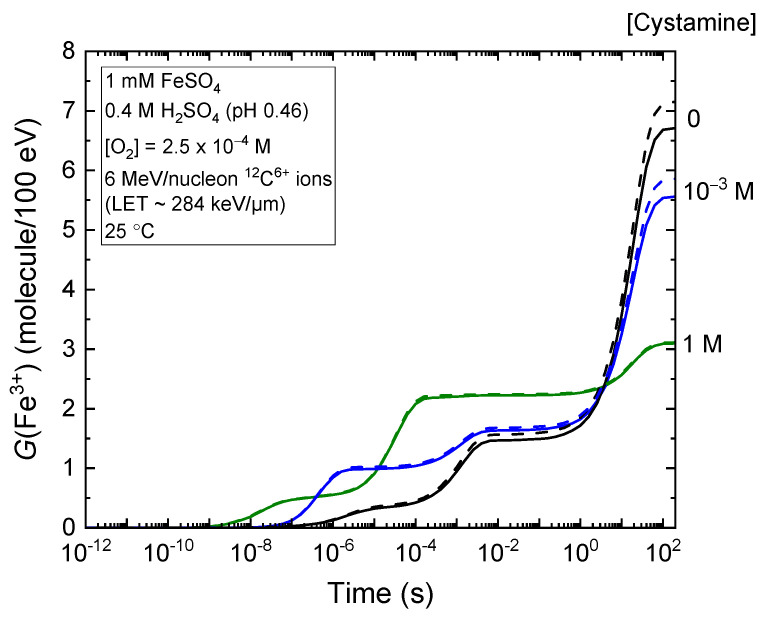
Temporal dependence of *G*(Fe^3+^) (expressed in molecules per 100 eV) of the radiolysis of aerated Fricke–cystamine solutions (containing 1 mM of FeSO_4_ in aqueous 0.4 M H_2_SO_4_) with varying concentrations of cystamine at 25 °C, over the time interval of ~1 ps to 200 s. We used incident 6 MeV per nucleon ^12^C^6+^ ions, corresponding to an LET value of ~248 keV/μm. The solid lines represent the results of our simulations that include the mechanism of multiple ionization of water: no added cystamine is shown with a black line, 10^−3^ M cystamine with a blue line, and 1 M cystamine with an olive line. These concentrations are denoted on the figure’s right side. For comparison, the dashed lines correspond to the same calculations but without considering MI. Note that currently no experimental data exist for a direct comparison with our results.

Figure 5 shows our calculated kinetics of Fe^3+^ formation during the radiolysis of aerated Fricke–cystamine solutions subjected to 6 MeV per nucleon carbon ions at 25 °C, spanning the time range of ~1 ps to 200 s after ionization. These are presented for different concentrations of cystamine: 0, 10^−3^, and 1 M selected for illustration purposes. Solid lines depict the outcomes from our simulations that incorporate the MI mechanism of water, while dashed lines represent those without the inclusion of MI (as depicted in Figure 1d).

**Figure 6:**
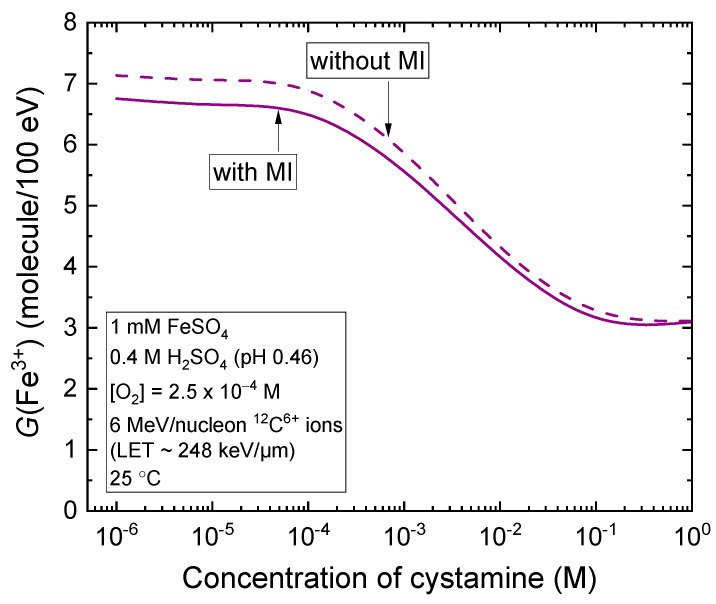
Dependence of the Fe^3+^ ion yield (expressed in molecules per 100 eV), obtained from our Monte Carlo simulations of the 6 MeV per nucleon carbon ion radiolysis of aerated Fricke–cystamine solutions at ~200 s after ionization, upon the concentration of added cystamine, which ranged from 10^−6^ to 1 M. The solid line represents the results of our simulations that include the mechanism of multiple ionization of water. For comparison, the dashed line represents the same calculations but without taking MI into account.

As can be seen, incorporating the multiple ionization of water in the simulations tends to lower the Fricke *G* values. For instance, at a concentration of 10^−3^ M cystamine, *G*(Fe^3+^), expressed as molecules per 100 eV, decreases from 5.86 without MI to 5.56 with MI, reflecting a reduction of about 5%. As the concentration of cystamine increases, this decrease in *G*(Fe^3+^) becomes increasingly less pronounced, and it is almost negligible at 1 M. Figure 6 further illustrates these results by comparing the impact of varying cystamine concentrations, ranging from 10^−6^ to 1 M, on *G*(Fe^3+^) during the 6 MeV per nucleon carbon ion radiolysis of Fricke–cystamine solutions, both with and without MI of water molecules.

The decrease in Fe^3+^ ion formation upon the incorporation of MI of water molecules is largely due to the marked reduction in H_2_O_2_ yield (see Equation (5)), as seen earlier in the radiolysis of liquid water by carbon ions at LETs greater than ~180 keV/μm (see Figure 3 of Meesungnoen and Jay-Gerin [30]). Given that H_2_O_2_ is formed almost entirely within the tracks through the combination of two ^•^OH radicals:

^•^OH + ^•^OH → H_2_O_2_, (3)

the reduction in *g*(H_2_O_2_) can be attributed to a lowered initial yield of ^•^OH, a result of Reaction (1), which does not generate ^•^OH radicals, thereby overshadowing the proton transfer reaction:

H_2_O^+^ + H_2_O → H_3_O^+^ + ^•^OH, (4)

which takes place after the formation of singly ionized water molecules. An additional factor contributing to the reduced H_2_O_2_ yield, in 248 keV/μm ^12^C^6+^ tracks, is the competition between Reactions (2) and (3).

## 3. Materials and Methods

### 3.1. The Ferrous Sulfate, or Fricke, Chemical Dosimeter

In chemical dosimetry, the dose is determined by measuring the chemical changes induced by radiation in a suitable medium. Any well-characterized quantitative chemical reaction can serve as the basis for a dosimeter. One of the most extensively studied systems in radiation chemistry, and arguably the best understood, is the air-saturated (~2.5 × 10^−4^ M O_2_) solution of 1 mM of ferrous sulfate in aqueous 0.4 M H_2_SO_4_ (pH~0.46) commonly referred to as the (standard) “Fricke dosimeter” [20,52,53]. The chemistry of this system is based upon the oxidation of Fe^2+^ to Fe^3+^ ions, driven by oxidizing species, such as ^•^OH, HO_2_^•^ (primarily because of the very rapid conversion of e^−^_aq_ to H^•^ at low pH and to HO_2_^•^ in the presence of oxygen), and H_2_O_2_, which arise from the radiolytic decomposition of (acidic) water [19,20,21,22,23,79].

Summing all sources of Fe^3+^ ions (e.g., see Table 1 (Reactions (1)–(8)) of Sepulveda et al. [14]; details are provided below), the yield of Fe^3+^ ions in an irradiated Fricke dosimeter can be expressed in terms of the primary (or escape) yields of radical and molecular species resulting from the radiolysis of the solution by the following stoichiometric equation (e.g., see [20,90]):

*G*(Fe^3+^) = 3 [*g*(H^•^) + *g*(HO_2_^•^)] + *g*(^•^OH) + 2 *g*(H_2_O_2_). (5)

For ^60^Co γ-irradiated 0.4 M H_2_SO_4_ aqueous solutions at room temperature, *g*(H^•^) = 3.70, *g*(^•^OH) = 2.90, *g*(H_2_O_2_) = 0.80, and *g*(HO_2_^•^) = 0.02 [22]. Using these primary yield values in Equation (5) gives a value of *G*(Fe^3+^) which is well within the range of 1–2% of the experimentally observed Fe^3+^ ion yield of 15.5 ± 0.2 ions per 100 eV for ^60^Co γ-rays, i.e., for radiation tracks consisting only of isolated spurs (LET~0.3 keV/μm) [20,53,91,92,93,94]. Because of its accuracy, reproducibility, and the linearity of its response to absorbed dose, the Fricke dosimeter is commonly used in radiation chemical work [91,92].

Equation (5) indicates that Fe^3+^ ion production is greatly affected by factors altering the primary radical yields, especially the yield of H^•^ atoms, which, in the presence of 0.4 M H_2_SO_4_, combines the yields of H^•^ generated directly by radiolysis and those formed by the protonation of e^−^_aq_. A key factor is the LET, which defines the radiation beam quality. Data from a number of authors have shown that *G*(Fe^3+^) consistently decreases as LET increases (see, for instance, [14,20,75,76,77,78,79,90]). This trend primarily stems from the enhanced role of radical–radical recombination reactions that yield molecular products—a characteristic inherent to the dense ionizing nature of high-LET radiation. Essentially, high-LET radiation lowers the number of free radicals that can migrate from the tracks to oxidize ferrous ions.

Interestingly, high-dose-rate radiation also reduces *G*(Fe^3+^) similarly to high-LET radiation [15,20,93,95,96], though their mechanisms of action are distinct. For high dose rates, there is an increased probability of radical–radical reactions (involving the H^•^, ^•^OH, and HO_2_^•^ radicals either with themselves or with one another) in the bulk of the solution due to *intertrack* reactions. In contrast, during high-LET irradiations, this probability increases within individual radiation tracks because of *intratrack* reactions [61].

### 3.2. Monte Carlo Track Chemistry Simulations of the Radiolysis of Fricke–Cystamine Solutions Using High-Energy Carbon Ions

To simulate the radiolysis of aerated Fricke–cystamine solutions at 25 °C with high-energy carbon ion irradiation, we used our Monte Carlo track chemistry computer code, IONLYS-IRT [58,59,60]. As this code has been described in detail elsewhere [13,14], we will offer only a brief summary of its primary features below.

Our “IONLYS” step-by-step program initially simulates the early *physical and physicochemical stages* of radiation action up to ~1 ps in track development within a 3D geometric environment. The intricate and highly nonhomogeneous spatial distribution of reactants generated by this program then directly serves as the starting point for the subsequent *chemical stage*, which occurs after 1 ps. In this third stage, the different radiolytic species diffuse randomly at rates determined by their diffusion coefficients, reacting with each other or, competitively, with dissolved solutes, such as oxygen in aerated Fricke solution or cystamine in the present study. This stage is covered by our “IRT” program [59], which uses the “independent reaction times” (IRT) method [97,98], a computationally efficient stochastic simulation technique that calculates reaction times without tracking the trajectories of each diffusing species. The ability of this method to generate reliable time-dependent chemical yields has been thoroughly validated over a broad spectrum of irradiation conditions by comparison with full random flight Monte Carlo simulations that do follow reactant trajectories in detail [99]. It is also important to highlight that our IRT program can describe reactions that occur over extended periods of time, well after the tracks have dissipated and when the radiolytic products are homogeneously distributed throughout the solution. This is especially relevant to simulations of the radiolysis of the Fricke dosimeter, where the Fe^3+^ ions are generated at various moments up to ~200 s (see, e.g., Figure 1 of Meesat et al. [13]) [13,14,15,90,100].

In simulating the radiolysis of Fricke–cystamine solutions, we employed in our IRT program the same chemical reaction scheme, rate constants, and diffusion coefficients for the reactive species as those in previous studies [13,14,15]. In summary, we added to the radiolysis reaction scheme for pure liquid water (as detailed in [23,59,60,90]) the reactions from Table 7 of Tippayamontri et al. [90], which account for species, such as HSO_4_^−^, SO_4_^2−^, SO_4_^•−^, and S_2_O_8_^2−^, present in irradiated 0.4 M H_2_SO_4_ aqueous solutions. To model the chemistry of the Fricke dosimeter in the IRT program, we incorporated the reactions involving the Fe^2+^ ions and the oxidizing species ^•^OH, HO_2_^•^, and H_2_O_2_ that are produced in the water of irradiated solutions under aerated conditions, namely:

^•^OH + Fe^2+^ → Fe^3+^ + OH^−^ *k* = 3.4 × 10^8^ M^−1^ s^−1^ (6)

HO_2_^•^ + Fe^2+^ → Fe^3+^ + HO_2_^−^ *k* = 7.9 × 10^5^ M^−1^ s^−1^ (7)

H_2_O_2_ + Fe^2+^ → Fe^3+^ + ^•^OH + OH^−^ *k* = 52 M^−1^ s^−1^ (8)

H^•^ + Fe^2+^ (+ H^+^) → Fe^3+^ + H_2_ *k* = 1.3 × 10^7^ M^−1^ s^−1^, (9)

where the rate constants (*k*) for the individual reactions are well established at 25 °C (see Table 1 of Sepulveda et al. [14]). Note that while some H^•^ atoms might directly interact with Fe^2+^ via Reaction (9), the influence of this reaction on Fe^3+^ formation in an aerated solution with a 1 mM of ferrous ion concentration is minimal and can be overlooked. Moreover, under the irradiation conditions of our study, concentrations of radiolytic products are relatively low compared to the background levels of H^+^ (~0.4 M), Fe^2+^ ions (1 mM), and O_2_ (~2.5 × 10^−4^ M) in the solution. As a result, their reactions can be treated as pseudo-first-order within the IRT program.

Lastly, to simulate the radiolysis of aerated Fricke solutions supplemented with cystamine (RSSR), we included the twenty-seven chemical reactions from Table 2 of Meesat et al. [13]. Among these reactions, those most important for Fe^3+^ production are [13,14]:

RSSR + e^−^_aq_ → (RSSR)^•−^ *k* = 4.1 × 10^10^ M^−1^ s^−1^ (10)

RSSR + H^•^ → RS^•^ + RSH *k* = 8 × 10^9^ M^−1^ s^−1^ (11)

RSSR + ^•^OH (+ H^+^) → (RSSR)^•+^ + H_2_O *k* = 1.7 × 10^10^ M^−1^ s^−1^ (12)

(RSSR)^•−^ + H^+^ → RS^•^ + RSH *k* = 4.2 × 10^9^ M^−1^ s^−1^ (13)

RS^•^ + O_2_ → RSOO^•^ *k* = 2 × 10^9^ M^−1^ s^−1^ (14)

RSOO^•^ + Fe^2+^ (+ H^+^) → Fe^3+^ + RSOOH *k* = 10^7^ M^−1^ s^−1^ (15)

RS^•^ + Fe^2+^ → Fe^3+^ + RS^−^ *k* = 2.5 × 10^8^ M^−1^ s^−1^ (16)

RS^•^ + RSSR → RSSSR + R^•^ *k* = 10^6^ M^−1^ s^−1^ (17)

R^•^ + O_2_ → ROO^•^ *k* = 2 × 10^9^ M^−1^ s^−1^ (18)

ROO^•^ + Fe^2+^ (+ H^+^) → Fe^3+^ + ROOH *k* = 7.9 × 10^5^ M^−1^ s^−1^ (19)

(RSSR)^•+^ + Fe^2+^ → Fe^3+^ + RSSR *k* = 2 × 10^6^ M^−1^ s^−1^ (20)

(RSSR)^•+^ + (RSSR)^•+^ → (RSSR)^2+^ + RSSR *k* = 2.5 × 10^9^ M^−1^ s^−1^, (21)

where the rate constants mentioned here for reactions between ions are derived from conditions of infinite dilution, where no ion–ion interactions take place. In fact, within the IRT program, we accounted for effects stemming from the ionic strength of the solutions in all reactions between ions. The only exception is the self-recombination of e^−^_aq_, for which there is no apparent evidence from ionic strength effect [101]. We adjusted the reaction rate constants for ionic strength using the procedure previously used by Sepulveda et al. [14].

Additionally, we did not account for the “direct” action of ionizing radiation on the solutes present in the solution. Given the concentrations of H_2_SO_4_ (0.4 M), ferrous ions (1 mM), dissolved oxygen (2.5 × 10^−4^ M), and cystamine (ranging from 10^−6^ M to 1 M), this approximation seems reasonable [13,14]. For instance, in a 0.4 M H_2_SO_4_ solution, only a small fraction (3.6%) of the total energy deposition is initially absorbed by the H_2_SO_4_^−^ ions. This fraction increases to 14.7% for a 1 M cystamine solution, which is the highest RSSR concentration we studied.

It is worth highlighting that our proposed reaction model for the aqueous radiation chemistry of cystamine enabled us to precisely match—without resorting to any free adjustable parameters—the experimental yields of Fe^3+^ observed in irradiated Fricke–cystamine solutions with X- and ^60^Co γ-rays [13,50,51]. This accuracy was consistent across a broad range of cystamine concentrations, irrespective of the presence or absence of oxygen [13,14,15]. Such a consistency between our simulated *G*(Fe^3+^) values and those observed underscores the validity of the overall reaction scheme we adopted to describe the radiation chemistry of cystamine in Fricke solutions.

We performed all calculations by simulating short (~1.5–30 μm) ^12^C^6+^ ion track segments. Within these segments, the energy (6–500 MeV per nucleon) and LET (~248–9.3 keV/μm) of the incident ion are well defined and stay nearly unchanged. Depending on the irradiating ion energy, typically 5000 to 100,000 reactive chemical species are generated within these simulated track segments. This not only ensures minimal statistical variations in calculating average chemical yields but also keeps computational durations within reasonable limits.

## 4. Conclusions

In this study, we examined the radical scavenging capability of cystamine from a purely radiation chemical perspective. We specifically studied its interaction with respect to the primary chemical species formed during the radiolysis of the ferrous sulfate (Fricke) dosimeter when subjected to high-energy carbon ions of varying linear energy transfer (LET) values. As a measurable indicator of cystamine’s radioprotective effectiveness, we employed the radiolytic oxidation of Fe^2+^ to Fe^3+^ ions in Fricke solutions. Our objective was to delve deeper into the underlying molecular mechanisms driving cystamine’s actions under 6–500 MeV per nucleon carbon ion irradiation. This encompasses LET values from ~248 to 9.3 keV/μm, and we investigated a wide range of cystamine concentrations from 10^−6^ to 1 M. The radiation-induced chemistry of the Fricke–cystamine solutions was simulated at 25 °C under aerated conditions, employing our Monte Carlo track chemistry computer code IONLYS-IRT.

In our simulations, we initially demonstrated that the marked reduction in *G*(Fe^3+^) at our lowest cystamine concentration (10^−6^ M) with increasing LET primarily arose from the increased importance of intratrack radical–radical processes in denser ionizing radiations. Indeed, these processes decrease the number of radicals escaping from the tracks, subsequently reducing the occurrences of radical–Fe^2+^ reactions and, in turn, *G*(Fe^3+^). This observation closely aligns with many experimental findings in the literature that address the influence of LET on the chemistry and yields of the Fricke dosimeter.

We then confirmed the ability of cystamine to neutralize free radicals generated during the irradiation of neighboring water, highlighting its robust antioxidant properties. Specifically, we found that the oxidation of Fe^2+^ ions to Fe^3+^ mainly happens via reactions with HO_2_^•^, H_2_O_2_, and radicals originating from cystamine, namely RS^•^ and RSSR^•+^.

The observed decrease in *G*(Fe^3+^) could be traced back to two synergistic radioprotective effects: one largely influenced by LET (especially evident at cystamine concentrations below ~10^−4^ M), and the other from cystamine’s presence (more dominant at higher concentrations). Based on our analysis, we concluded in particular that at reduced concentrations, cystamine offers only limited protection at high LET, aligning with findings from earlier studies. Conversely, at cystamine concentrations beyond ~10^−4^ M, a significant decrease in *G*(Fe^3+^) was observed, regardless of the LET value. This marked reduction suggested that at these concentrations, the radioprotection of Fe^2+^ ions during the radiolysis of Fricke–cystamine solutions is driven more by the presence of cystamine than the LET. To account for these findings, we proposed that cystamine increasingly interacts with carbon ion tracks prior to their complete expansion. In these conditions, cystamine scavenges early-formed free radicals within the columnar regions of the tracks, competing with inter–radical combination reactions. This, in turn, reduces the number of radicals available for Fe^2+^ ion oxidation. At even greater concentrations of cystamine (exceeding ~10^−3^ M), these cystamine–radical reactions become increasingly prevalent, substantially reducing the influence of LET at these levels. Ultimately, when the cystamine concentration surpasses ~0.1 M, *G*(Fe^3+^) was found to stay largely unchanged regardless of the LET value, suggesting that the compound might not neutralize all the initially formed radicals in high-LET carbon ion tracks rapidly enough to effectively counter the radical–radical combination reactions.

Finally, we examined the influence of very high LETs on cystamine’s radioprotective and antioxidant capabilities, with particular attention to the multiple (primarily double) ionization (MI) of water molecules. This is especially pertinent for 6 MeV per nucleon carbon ion irradiation, where the LET reaches ~248 keV/μm. Incorporating MI into the simulations yielded Fricke *G* values approximately 5% lower than those obtained without MI consideration. Our analysis indicates that this decrease is largely due to a significant reduction in the H_2_O_2_ yield during the track stage of radiolysis.

In summary, these study’s results carry substantial predictive significance. The alignment between the calculated yields and the available measured values, achieved without requiring adjustable parameters, strongly validates the kinetic schemes, reaction rate constants, diffusion coefficients, and pathway parameters utilized in our Monte Carlo track chemistry computational approach. This approach effectively portrays the radiolysis of cystamine in aerated Fricke solutions when exposed to 6–500 MeV per nucleon carbon ion irradiation. Furthermore, assuming that our fundamental research findings at pH 0.46 are applicable to biological systems operating at a physiological pH, incorporating cystamine into carbon ion hadrontherapy could be a promising approach to further enhance therapeutic efficacy in cancer treatments. Specifically, in carbon ion therapy, the entrance channel or “plateau” region of the ion depth–dose distribution, corresponding to “normal” tissue, exhibits a low LET. Cystamine significantly protects these tissues from radiation in this region. Conversely, in the “Bragg peak” region, at the end of the ion’s range within the “tumor” volume, the radiation’s LET is elevated. This increased LET diminishes the effectiveness of cystamine, in turn enhancing cell-killing efficacy.

## Data Availability

Data generated or analyzed during this study are provided in full within the article.

## References

[1] Halperin E.C., Wazer D.E., Perez C.A., Brady L.W. (2019). Perez & Brady’s Principles and Practice of Radiation Oncology.

[2] Baskar R., Lee K.A., Yeo R., Yeoh K.-W. (2012). Cancer and radiation therapy: Current advances and future directions. Int. J. Med. Sci..

[3] Smith T.A., Kirkpatrick D.R., Smith S., Smith T.K., Pearson T., Kailasam A., Herrmann K.Z., Schubert J., Agrawal D.K. (2017). Radioprotective agents to prevent cellular damage due to ionizing radiation. J. Transl. Med..

[4] Hall E.J., Giaccia A.J. (2019). Radiobiology for the Radiologist.

[5] Poggi M.M., Coleman C.N., Mitchell J.B. (2001). Sensitizers and protectors of radiation and chemotherapy. Curr. Probl. Cancer.

[6] Weiss J.F., Landauer M.R. (2009). History and development of radiation-protective agents. Int. J. Radiat. Biol..

[7] Kamran M.Z., Ranjan A., Kaur N., Sur S., Tandon V. (2016). Radioprotective agents: Strategies and translational advances. Med. Res. Rev..

[8] Nair C.K.K., Parida D.K., Nomura T. (2001). Radioprotectors in radiotherapy. J. Radiat. Res..

[9] Johnke R.M., Sattler J.A., Allison R.R. (2014). Radioprotective agents for radiation therapy: Future trends. Future Oncol..

[10] Bump E.A., Malaker K. (1998). Radioprotectors: Chemical, Biological, and Clinical Perspectives.

[11] Conklin J.J., Walker R.I. (1987). Military Radiobiology.

[12] Dziegielewski J., Goetz W., Baulch J.E. (2010). Heavy ions, radioprotectors and genomic instability: Implications for human space exploration. Radiat. Environ. Biophys..

[13] Meesat R., Sanguanmith S., Meesungnoen J., Lepage M., Khalil A., Jay-Gerin J.-P. (2012). Utilization of the ferrous sulfate (Fricke) dosimeter for evaluating the radioprotective potential of cystamine: Experiment and Monte Carlo simulation. Radiat. Res..

[14] Sepulveda E., Sanguanmith S., Meesungnoen J., Jay-Gerin J.-P. (2019). Evaluation of the radioprotective ability of cystamine for 150 keV–500 MeV proton irradiation: A Monte Carlo track chemistry simulation study. Can. J. Chem..

[15] Penabeï S., Meesungnoen J., Jay-Gerin J.-P. (2023). Assessment of cystamine’s radioprotective/antioxidant ability under high-dose-rate irradiation: A Monte Carlo multi-track chemistry simulation study. Antioxidants.

[16] von Sonntag C. (2006). Free-Radical-Induced DNA Damage and Its Repair: A Chemical Perspective.

[17] Cadet J., Davies K.J.A., Medeiros M.H., Di Mascio P., Wagner J.R. (2017). Formation and repair of oxidatively generated damage in cellular DNA. Free Radic. Biol. Med..

[18] Azzam E.I., Jay-Gerin J.-P., Pain D. (2012). Ionizing radiation-induced metabolic oxidative stress and prolonged cell injury. Cancer Lett..

[19] Buxton G.V., Farhataziz, Rodgers M.A.J. (1987). Radiation chemistry of the liquid state: (1) Water and homogeneous aqueous solutions. Radiation Chemistry: Principles and Applications.

[20] Spinks J.W.T., Woods R.J. (1990). An Introduction to Radiation Chemistry.

[21] Ferradini C., Jay-Gerin J.-P. (1999). Radiolysis of water and aqueous solutions: History and present state of the science. Can. J. Chem..

[22] Ferradini C., Jay-Gerin J.-P. (2000). The effect of pH on water radiolysis: A still open question—A minireview. Res. Chem. Interm..

[23] Elliot A.J., Bartels D.M. (2009). The Reaction Set, Rate Constants and g-Values for the Simulation of the Radiolysis of Light Water over the Range 20 to 350 °C Based on Information Available in 2008.

[24] Klassen N.V. (1991). Primary species in irradiated water. J. Chim. Phys..

[25] Bielski B.H.J., Cabelli D.E., Arudi R.L., Ross A.B. (1985). Reactivity of HO_2_/O_2_^−^ radicals in aqueous solution. J. Phys. Chem. Ref. Data.

[26] Kuppermann A., Haïssinsky M. (1961). Diffusion kinetics in radiation chemistry. Actions Chimiques et Biologiques des Radiations.

[27] Linear Energy Transfer (1970).

[28] Mozumder A. (1999). Fundamentals of Radiation Chemistry.

[29] LaVerne J.A., Mozumder A., Hatano Y. (2004). Radiation chemical effects of heavy ions. Charged Particle and Photon Interactions with Matter: Chemical, Physicochemical, and Biological Consequences with Applications.

[30] Meesungnoen J., Jay-Gerin J.-P. (2005). High-LET radiolysis of liquid water with ^1^H^+^, ^4^He^2+^, ^12^C^6+^, and ^20^Ne^9+^ ions: Effects of multiple ionization. J. Phys. Chem. A.

[31] Magee J.L. (1953). Radiation chemistry. Annu. Rev. Nucl. Sci..

[32] Freeman G.R., Baxendale J.H., Busi F. (1982). Basics of radiation chemistry. The Study of Fast Processes and Transient Species by Electron Pulse Radiolysis: Proceedings of the NATO Advanced Study Institute Held at Capri, Italy, 7–18 September 1981.

[33] Chatterjee A., Holley W.R. (1993). Computer simulation of initial events in the biochemical mechanisms of DNA damage. Adv. Radiat. Biol..

[34] Sanguanmith S., Meesungnoen J., Muroya Y., Lin M., Katsumura Y., Jay-Gerin J.-P. (2012). On the spur lifetime and its temperature dependence in the low linear energy transfer radiolysis of water. Phys. Chem. Chem. Phys..

[35] Muroya Y., Plante I., Azzam E.I., Meesungnoen J., Katsumura Y., Jay-Gerin J.-P. (2006). High-LET ion radiolysis of water: Visualization of the formation and evolution of ion tracks and relevance to the radiation-induced bystander effect. Radiat. Res..

[36] Boscolo D., Krämer M., Fuss M.C., Durante M., Scifoni E. (2020). Impact of target oxygenation on the chemical track evolution of ion and electron radiation. Int. J. Mol. Sci..

[37] Ward J.F., Nygaard O.F., Simić M.G. (1983). Chemical aspects of DNA radioprotection. Radioprotectors and Anticarcinogens.

[38] Bacq Z.-M., Alexander P. (1955). Principes de Radiobiologie.

[39] Bacq Z.-M., Beaumariage M.L. (1965). Action radioprotectrice de la cystéamine et de la cystamine chez la souris en fonction du temps séparant l’injection du protecteur du début de l’irradiation par rayons X. Arch. Int. Pharmacodyn. Ther..

[40] Pinto J.T., Van Raamsdonk J.M., Leavitt B.R., Hayden M.R., Jeitner T.M., Thaler H.T., Krasnikov B.F., Cooper A.J. (2005). Treatment of YAC128 mice and their wild-type littermates with cystamine does not lead to its accumulation in plasma or brain: Implications for the treatment of Huntington disease. J. Neurochem..

[41] Jeitner T.M., Pinto J.T., Cooper A.J.L. (2018). Cystamine and cysteamine as inhibitors of transglutaminase activity in vivo. Biosci. Rep..

[42] Bousquet M., Gibrat C., Ouellet M., Rouillard C., Calon F., Cicchetti F. (2010). Cystamine metabolism and brain transport properties: Clinical implications for neurodegenerative diseases. J. Neurochem..

[43] Paul B.D., Snyder S.H. (2019). Therapeutic applications of cysteamine and cystamine in neurodegenerative and neuropsychiatric diseases. Front. Neurol..

[44] Jayson G.G., Owen T.C., Wilbraham A.C. (1967). The radiation chemistry of cystamine sulphate. J. Chem. Soc. B Phys. Org..

[45] Bidzilya V.A., Golovkova L.P., Beregovskaya N.N., Basyuk V.V., Korol’ É.N., Chuiko A.A., Znamenskii V.V., Barkaya V.S. (1991). Radioprotective effect of immobilized cystamine. Pharm. Chem. J..

[46] Tremblay M.-È., Saint-Pierre M., Bourhis E., Lévesque D., Rouillard C., Cicchetti F. (2006). Neuroprotective effects of cystamine in aged parkinsonian mice. Neurobiol. Aging.

[47] Borrell-Pagès M., Canals J.M., Cordelières F.P., Parker J.A., Pineda J.R., Grange G., Bryson E.A., Guillermier M., Hirsch E., Hantraye P. (2006). Cystamine and cysteamine increase brain levels of BDNF in Huntington disease via HSJ1b and transglutaminase. J. Clin. Investig..

[48] Okauchi M., Xi G., Keep R.F., Hua Y. (2009). Tissue-type transglutaminase and the effects of cystamine on intracerebral hemorrhage-induced brain edema and neurological deficits. Brain Res..

[49] Toohey J.I. (2009). Sulfur metabolism in AIDS: Cystamine as an anti-HIV agent. AIDS Res. Hum. Retroviruses.

[50] Jayson G.G., Wilbraham A.C. (1968). The utilisation of the Fricke dosimeter for evaluating the biological radiation-protective potential of water-soluble organic compounds. Chem. Commun..

[51] Lalitha B., Mittal J.P. (1971). Electron transfer reaction in the radiation chemistry of some biologically important disulphide compounds. Radiat. Eff..

[52] Fricke H., Morse S. (1927). The chemical action of roentgen rays on dilute ferrosulphate solutions as a measure of dose. Am. J. Roentgenol. Radium Ther..

[53] Fricke H., Hart E.J., Attix F.H., Roesch W.C. (1966). Chemical dosimetry. Radiation Dosimetry.

[54] Dewhurst H.A. (1951). Effect of organic substances on the γ-ray oxidation of ferrous sulfate. J. Chem. Phys..

[55] Guczi L. (1962). Étude de l’effet d’addition de diverses substances sur l’oxydation des ions ferreux en solution aqueuse. J. Chim. Phys..

[56] Das R.C. (1971). Radiation chemistry of aqueous aerated ferrous sulphate solution. Radiat. Res. Rev..

[57] Matthews R.W. (1982). Aqueous chemical dosimetry. Int. J. Appl. Radiat. Isot..

[58] Cobut V., Frongillo Y., Patau J.P., Goulet T., Fraser M.-J., Jay-Gerin J.-P. (1998). Monte Carlo simulation of fast electron and proton tracks in liquid water. I. Physical and physicochemical aspects. Radiat. Phys. Chem..

[59] Frongillo Y., Goulet T., Fraser M.-J., Cobut V., Patau J.P., Jay-Gerin J.-P. (1998). Monte Carlo simulation of fast electron and proton tracks in liquid water. II. Nonhomogeneous chemistry. Radiat. Phys. Chem..

[60] Meesungnoen J., Jay-Gerin J.-P., Hatano Y., Katsumura Y., Mozumder A. (2011). Radiation chemistry of liquid water with heavy ions: Monte Carlo simulation studies. Charged Particle and Photon Interactions with Matter: Recent Advances, Applications, and Interfaces.

[61] Alanazi A., Meesungnoen J., Jay-Gerin J.-P. (2021). A computer modeling study of water radiolysis at high dose rates. Relevance to FLASH radiotherapy. Radiat. Res..

[62] Favaudon V., Caplier L., Monceau V., Pouzoulet F., Sayarath M., Fouillade C., Poupon M.F., Brito I., Hupé P., Bourhis J. (2014). Ultrahigh dose-rate FLASH irradiation increases the differential response between normal and tumor tissue in mice. Sci. Transl. Med..

[63] Esplen N., Mendonca M.S., Bazalova-Carter M. (2020). Physics and biology of ultrahigh dose-rate (FLASH) radiotherapy: A topical review. Phys. Med. Biol..

[64] Limoli C.L., Vozenin M.-C. (2023). Reinventing radiobiology in the light of FLASH radiotherapy. Annu. Rev. Cancer Biol..

[65] Burns P.C., Ewing R.C., Navrotsky A. (2012). Nuclear fuel in a reactor accident. Science.

[66] Sokol O., Durante M. (2023). Carbon ions for hypoxic tumors: Are we making the most of them?. Cancers.

[67] Castro J.R. (1995). Results of heavy ion radiotherapy. Radiat. Environ. Biophys..

[68] Schlaff C.D., Krauze A., Belard A., O’Connell J.J., Camphausen K.A. (2014). Bringing the heavy: Carbon ion therapy in the radiobiological and clinical context. Radiat. Oncol..

[69] Mohamad O., Makishima H., Kamada T. (2018). Evolution of carbon ion radiotherapy at the National Institute of Radiological Sciences in Japan. Cancers.

[70] Malouff T.D., Mahajan A., Krishnan S., Beltran C., Seneviratne D.S., Trifiletti D.M. (2020). Carbon ion therapy: A modern review of an emerging technology. Front. Oncol..

[71] Pompos A., Foote R.L., Koong A.C., Le Q.T., Mohan R., Paganetti H., Choy H. (2022). National effort to re-establish heavy ion cancer therapy in the United States. Front. Oncol..

[72] Schardt D., Elsässer T., Schulz-Ertner D. (2010). Heavy-ion tumor therapy: Physical and radiobiological benefits. Rev. Mod. Phys..

[73] Tinganelli W., Durante M. (2020). Carbon ion radiobiology. Cancers.

[74] Amaldi U., Kraft G. (2005). Radiotherapy with beams of carbon ions. Rep. Prog. Phys..

[75] Christman E.A., Appleby A., Jayko M. (1981). Radiation chemistry of high-energy carbon, neon, and argon ions: Integral yields from ferrous sulfate solutions. Radiat. Res..

[76] LaVerne J.A., Schuler R.H. (1994). Track effects in water radiolysis: Yields of the Fricke dosimeter for carbon ions with energies up to 1700 MeV. J. Phys. Chem..

[77] LaVerne J.A. (2000). Track effects of heavy ions in liquid water. Radiat. Res..

[78] Pimblott S.M., LaVerne J.A. (2002). Effects of track structure on the ion radiolysis of the Fricke dosimeter. J. Phys. Chem. A.

[79] Allen A.O. (1961). The Radiation Chemistry of Water and Aqueous Solutions.

[80] Barendsen G.W., Walter H.M.D. (1964). Effects of different ionizing radiations on human cells in tissue culture. IV. Modification of radiation damage. Radiat. Res..

[81] Wolff R.K., Aldrich J.E., Penner T.L., Hunt J.W. (1975). Picosecond pulse radiolysis. V. Yield of electrons in irradiated aqueous solution with high concentrations of scavenger. J. Phys. Chem..

[82] Bevan P.L.T., Hamill W.H. (1970). Evidence for very early ionic effects in water radiolysis. Trans. Faraday Soc..

[83] Ražem D., Dvornik I., Dobó J., Hedvig P. (1972). Scavenging of electrons prior to thermalization in ethanol. Proceedings of the Third Tihany Symposium on Radiation Chemistry.

[84] Sanguanmith S., Meesungnoen J., Muroya Y., Jay-Gerin J.-P. (2021). Scavenging of “dry” electrons prior to hydration by azide ions: Effect on the formation of H_2_ in the radiolysis of water by ^60^Co γ-rays and tritium β-electrons. Can. J. Chem..

[85] Gardès-Albert M., Jore D., Abedinzadeh Z., Rouscilles A., Deycard S., Bouffard S. (1996). Réduction du tétranitrométhane par les espèces primaires formées lors de la radiolyse de l’eau par des ions lourds Ar^18+^ (in French). J. Chim. Phys..

[86] Ferradini C., Jay-Gerin J.-P. (1998). Does multiple ionization intervene for the production of HO_2_^•^ radicals in high-LET liquid water radiolysis?. Radiat. Phys. Chem..

[87] Zakaria A.M., Colangelo N.W., Meesungnoen J., Azzam E.I., Plourde M.-É., Jay-Gerin J.-P. (2020). Ultra-high dose-rate, pulsed (FLASH) radiotherapy with carbon ions: Generation of early, transient, highly oxygenated conditions in the tumor environment. Radiat. Res..

[88] Meesungnoen J. (2007). Effect of Multiple Ionization on the Radiolysis of Liquid Water Irradiated with Heavy Ions: A Theoretical Study Using Monte Carlo Simulations. Ph.D. Thesis.

[89] Platzman R.L., Nickson J.J. (1952). On the primary processes in radiation chemistry and biology. Symposium on Radiobiology. The Basic Aspects of Radiation Effects on Living Systems.

[90] Tippayamontri T., Sanguanmith S., Meesungnoen J., Sunaryo G.R., Jay-Gerin J.-P., Pandalai S.G. (2009). Fast neutron radiolysis of the ferrous sulfate (Fricke) dosimeter: Monte Carlo simulations. Recent Research Developments in Physical Chemistry.

[91] Klassen N.V., Shortt K.R., Seuntjens J., Ross C.K. (1999). Fricke dosimetry: The difference between *G*(Fe^3+^) for ^60^Co γ-rays and high-energy X-rays. Phys. Med. Biol..

[92] McEwen M., El Gamal I., Mainegra-Hing E., Cojocaru C. (2014). Determination of the Radiation Chemical Yield (G) for the Fricke Chemical Dosimetry System in Photon and Electron Beams.

[93] (1982). The Dosimetry of Pulsed Radiation.

[94] (1969). Radiation Dosimetry: X rays and Gamma rays with Maximum Photon Energies Between 0.6 and 50 MeV.

[95] Sehested K., Bjergbakke E., Holm N.W., Fricke H. (1973). The reaction mechanism of the ferrous sulphate dosimeter at high dose rates. Dosimetry in Agriculture, Industry, Biology and Medicine.

[96] Precek M., Kubelik P., Vysin L., Schimdhammer U., Larbre J.-P., Demarque A., Jeunesse P., Mostafavi M., Juha L. (2022). Dose rate effects in fluorescence chemical dosimeters exposed to picosecond electron pulses: An accurate measurement of low doses at high dose rates. Radiat. Res..

[97] Tachiya M. (1983). Theory of diffusion-controlled reactions: Formulation of the bulk reaction rate in terms of the pair probability. Radiat. Phys. Chem..

[98] Pimblott S.M., Pilling M.J., Green N.J.B. (1991). Stochastic models of spur kinetics in water. Radiat. Phys. Chem..

[99] Plante I. (2009). Développement de Codes de Simulation Monte Carlo de la Radiolyse de l’Eau par des Électrons, Ions Lourds, Photons et Neutrons. Applications à Divers Sujets d’Intérêt Expérimental. Ph.D. Thesis.

[100] Bĕgusová M., Pimblott S.M. (2002). Stochastic simulation of γ radiolysis of acidic ferrous sulfate solution at elevated temperatures. Radiat. Prot. Dosim..

[101] Schmidt K.H., Bartels D.M. (1995). Lack of ionic strength effect in the recombination of hydrated electrons: (e^−^)_aq_ + (e^−^)_aq_ → 2(OH^−^) + H_2_. Chem. Phys..

